# A novel inhibitor of soluble epoxide hydrolase that adducts C521 is cardioprotective

**DOI:** 10.1016/j.redox.2025.103974

**Published:** 2025-12-11

**Authors:** Rebecca L. Charles, Mariana Fernandez-Caggiano, Olena Rudyk, Izaak Tyson-Hirst, Mazdak Ehteramyan, Christopher H. Switzer, Roberto Buccafusca, Vinothini Rajeeve, Katiuscia Bianchi, Valle Morales, Andrew J. Finch, Philip Eaton

**Affiliations:** a William Harvey Research Institute, Faculty of Medicine and Dentistry, Queen Mary University of London, London, UK; b School of Cardiovascular and Metabolic Medicine & Sciences, King's College London, London, UK; c Barts Cancer Institute, John Vane Science Centre, Queen Mary University of London, London, UK; d Department of Molecular and Cell Biology, University of Leicester, Leicester, UK; e School of Biological and Chemical Sciences, Queen Mary University of London, London, UK

**Keywords:** Soluble epoxide hydrolase, Inhibitor, Thiol, Heart, Ischemia and reperfusion

## Abstract

The lipid electrophile nitro-oleic acid (NO_2_-OA) and inhibitors of soluble epoxide hydrolase (sEH) limit injury during myocardial ischemia and reperfusion (IR). We investigated if cardioprotection by NO_2_-OA was mediated by inhibitory adduction of this electrophile to C521 of the hydrolase. Indeed, administering NO_2_-OA to wild type (WT) isolated perfused hearts prior to IR limited infarction, but this protection was absent in C521S sEH knock-in (KI) mice - demonstrating the critical importance of this cysteine. To identify more potent and selective inhibitors, we screened a library of electrophiles for their ability to inhibit sEH. A compound, we termed RLC14, had an IC_50_ of 6.8x10^−9^ M and protected WT, but not KI, isolated hearts from infarction during IR. Systemic administration of RLC14 decreased myocardial sEH activity and increased plasma EET/DHET ratio selectively in WT mice, consistent with inhibition of the hydrolase. In line with these findings, RLC14 protected WT, but not KI, mice from *in vivo* coronary artery ligation IR-induced infarction. Mass spectrometry analyses showed the novel electrophilic inhibitor, RLC14, which contains a disulfide, adducts to C521 in sEH to mediate its effects. This study identifies RLC14 as a potent cardioprotective agent that limits IR injury through C521-dependent hydrolase inhibition.

## 1 Introduction

Soluble epoxide hydrolase (sEH) catalyses the hydrolysis of epoxides such as EETs (epoxyeicosatrienoic acids) to their corresponding diols (dihydroxyeicosatrienoic acids, DHETs). EETs cause vasodilation and reduce blood pressure (BP) in hypertensive animals [[1], [2], [3], [4], [5]]. Consequently, sEH inhibition increases EETs abundance with consequent vasodilation, highlighting its importance in blood pressure regulation, an important determinant of cardiovascular health and disease [[6], [7], [8], [9], [10], [11], [12], [13]]. Inhibitors of sEH afford broad cardiovascular protection, including blockade of smooth muscle proliferation [14], reduction of atherosclerosis and hypertension [[15], [16], [17], [18], [19], [20]], prevention and regression of hypertrophy and heart failure [[21], [22], [23]], as well as limiting cardiac fibrosis [24]. sEH inhibition also limits ischemic damage in the heart [[25], [26], [27], [28], [29], [30]], brain and other organs [31,32]. Similarly, sEH-null mice are protected from cardiovascular pathologies [27] and genetic alterations that promote enhanced hydrolase activity are a risk factor for human heart failure [21].

We showed sEH activity is inhibited by electrophilic lipids, such as 15-deoxy-Δ^12,14^-prostaglandin J_2_ (15d-PGJ_2_) or nitro-oleic acid (NO_2_-OA) [[33], [34], [35]]. These endogenous lipid electrophiles bind and covalently adduct within the C-terminal hydrolase domain, inhibiting its catalytic activity to provide post-translational regulation of sEH. They adduct to Cys521 of sEH resulting in inhibition of its hydrolase activity, serving as ‘natures-own’ inhibitors of this enzyme. To investigate this further we generated a transgenic knock-in (KI) mouse in which the C521 in sEH was systemically replaced by a serine, making this mutant resistant to inhibition by endogenously derived or exogenously applied lipid electrophiles. NO_2_-OA administration was able to inhibit the hydrolase in wild type (WT), but not littermate in C521S sEH KI, mice. Consistent with this, administration of NO_2_-OA reduced MAP in angiotensin II-treated hypertensive WT mice, but was wholly ineffective in lowering blood pressure in littermate KIs [34]. This provided evidence that lipid electrophiles can target sEH and inhibit it by adducting to C521.

sEH is not only expressed in the vasculature, but also in other cardiovascular cells such as cardiomyocytes, consistent with conventional inhibitors of the hydrolase providing protection against myocardial IR injury [[25], [26], [27], [28], [29]]. We tested the rational hypothesis that NO_2_-OA would similarly limit myocardial injury during IR by inhibiting sEH in WT mice, but this cardioprotection would be deficient in C521S KI mice that lack the crucial cysteine to which the electrophile adducts. In addition, to identify structurally different electrophilic inhibitors that are potentially more potent and selective than NO_2_-OA, we screened a library of electrophilic molecules for their ability to inhibit sEH. We identified a new inhibitor of sEH, which we named RLC14, potently inhibited sEH activity, increased plasma EET/DHET ratio and attenuated myocardial infarction during IR in WT mice *in vivo*. This cardioprotection by RLC14 was absent in the KI mice, corroborating C521 as the target of this new sEH inhibitor.

## 2 Methods

### 2.1 Natural compound libraries

Compounds with potential electrophilic properties were obtained from AnalytiCon Discovery (plant-derived, microorganism derived and semi synthetic compounds) and InterBioscreen (derivatives of natural compounds, natural compounds and rare analogues) and are individually listed in Table S2.

### 2.2 Soluble epoxide hydrolase activity assay

A sEH inhibitor screening assay kit (Cayman) was used to assess potential sEH inhibitors from the compound libraries. Each compound was tested initially in duplicate at 10 μM. 20 compounds that attenuated activity by at least 90 % in the initial screen were then subjected to further testing to determine their IC_50_ for hydrolase activity.

### 2.3 Animal studies

All procedures were performed in accordance with the Home Office Guidance on the Operation of the Animals (Scientific Procedures) Act 1986 in the United Kingdom and were approved by the Queen Mary University of London animal welfare and ethical review body. Mice constitutively expressing C521S sEH were generated by Taconic and are on a background of C57BL/6 N Tac [34]. Age- and bodyweight –matched WT or C521S sEH KI male mice were studied. C57BL/6 mice were used for drug dosing studies.

### 2.4 *Ex vivo* ischemia and reperfusion

Adult male mice (25–30 g) were anesthetized by an intraperitoneal injection of pentobarbital sodium (300 mg/kg), mixed 50:50 with the anticoagulant heparin (150 units). The hearts were rapidly isolated, mounted onto a Langendorff apparatus, and retrogradely perfused at a constant pressure of 80 mmHg with Krebs-Henseleit buffer (KHB) containing (in mM) 118.5 NaCl, 25.0 NaHCO3, 4.75 KCl, 1.18 KH_2_PO4, 1.19 MgSO_4_, 11.0 d-glucose, and 1.4 CaCl_2_, equilibrated with 95 % O_2_, 5 % CO_2_ at 37 °C. A fluid-filled balloon inserted into the left ventricle cavity monitored contractile function and left ventricle developed pressure (LVDP). The balloon was gradually inflated until the left ventricular end-diastolic pressure (LVEDP) was between 4 and 10 mmHg. Atrial pacing was performed at 550–600 beats/min and coronary flow was measured by an electronic feedback circuit controlling the perfusion pump (STH Pump Controller, ADInstruments). The hearts were stabilised for 20 min followed by nitro-oleic acid (Cayman) or drug RLC14 perfusion for 20 min before ischemia. Global ischemia was induced by stopping perfusion for 30 min. Reperfusion (2 h) was initiated by raising flow back to the preischemic perfusion pressure (80 mmHg). Electrical pacing was stopped 2 min after contraction ceased during ischemia and was restarted 5 min into reperfusion. Heart rate developed pressure and coronary flow were measured.

### 2.5 Infarction assessment in isolated murine hearts

Infarct size was determined by 2,3,5 triphenyltetrazolium chloride (TTC) staining. After 2 h of reperfusion, hearts were perfused for 1 min with 5 ml of 3 % TTC in phosphate-buffered saline and then placed in an identical solution at 37 °C for 10 min. The atria were removed, the hearts were blotted dry, weighed and then stored at 80 °C for up to 1 week. The hearts were then semi-thawed and sectioned from apex to base in 1 mm slices using a Mouse Heart Slicer Matrix (Zivic Instruments). After sectioning, the slices were placed overnight in 10 % formaldehyde at room temperature before transferring to phosphate-buffered saline for an additional day at 4 °C. The sections were then compressed between glass plates (1 mm apart) and imaged (Epson Model G850A). After magnification, planimetry was carried out using image analysis software (Image J), and the surface areas of the whole and TTC-negative myocardium were transformed to volume by multiplication with tissue thickness. The TTC-negative infarction volume was expressed as a percentage of heart volume.

### 2.6 Administration of RLC14 to mice

C57/BL6 mice were anesthetised and subjected to subcutaneous implantation of osmotic mini-pumps (model 1002, Alzet) at an infusion rate of 0.5 mg/kg/day. The mini-pumps contained either RLC14, diluted in 50 % DMSO/50 % saline, drug treated group or 50 % DMSO/50 % saline only, control group. After 7 days, the hearts were rapidly isolated and frozen in liquid N_2_.

### 2.7 Soluble epoxide hydrolase activity assay in heart samples

Mouse hearts from animals that were treated with the drug RLC14 were rapidly isolated and frozen in liquid N_2_ at the end of the protocol. Hearts were powdered under and stored in liquid nitrogen until ready for analysis. They were homogenised (1 ml of buffer per 100 mg of cardiac tissue) on ice in 100 mM Tris-HCl, pH7.4 using a Polytron tissue grinder. Cytosolic fractions were prepared from the hearts by centrifugation at 25,000 g for 5 min at 4 °C. 20 μl of the cytosolic fraction was then used for the activity assay, adding it to 175 μl of 25 mM Tris-HCl pH 7.4. After equilibration at room temperature, the reaction was initiated by the addition of 5 μl of 10 μM 33-phenyl-cyano(6-methoxy-2-naphthalenyl)methyl ester-2-oxiraneacetic acid (PHOME, Cayman) dissolved in DMSO. The mixture was incubated for 15 min, followed by fluorescence measurement at 330/465 nm using a CLARIOstar plate reader (BMG Labtech). A standard curve using 6M2N was used for quantification purposes as described before [36]. In an additional reaction mixture, a sEH inhibitor (5 μl of 10 μM AUDA, Cayman) was added to ascertain the baseline reading for sEH activity for each sample.

### 2.8 *In vivo* ischemia and reperfusion

WT and C521S sEH KI mice were anesthetised and subjected to subcutaneous implantation of osmotic mini-pumps (model 1002, Alzet) containing either RLC14 (InterBioScreen) or saline at an infusion rate of 0.5 mg/kg/day. After 7 days, the mice were subjected to ischemia and reperfusion. Mice were placed inside an induction chamber where 4 % isoflurane was provided with an oxygen flow rate of 0.5 l/min until loss of righting reflex. Artificial ventilation of the lungs with 2 % isoflurane flow in 0.5 L/min oxygen was provided using a rodent ventilator (Hugo Sachs Elektronic MiniVent Type 845) set at 200 μl of stroke volume and at 150 strokes per minute. A rectal probe was inserted to monitor the temperature of the animal so that it could be maintained at 37 °C. Prior to surgery, the mice were injected with 200 μl of vetergesic (0.03 mg/ml). Surgery was carried out using a Leica Wild OP Mikroskop M650 Microscope (Leica) adjusted to 16 times magnification. The thorax was opened in the third intercostal room and the third and the fourth ribs were separated using microretractors in order to expose the operating region. An 8-0 nylon suture (Ethicon) was looped around the left anterior descending coronary artery (LAD). For temporary left ventricle ischemia, a 0.3 cm PE90 sterile tube was placed parallel to the LAD and a suture loop was tightened around it. The PE90 tube was released 30 min after ischemia and the loosened ligature was cut allowing reperfusion of the ventricle. Reperfusion was confirmed by the return of blood flow back into the left ventricle. The chest cavity, the layers of muscle and the skin were independently closed by interrupted stiches using 5-0 ethilon suture (Ethicon). Mice were disconnected from the ventilator as soon as the animals attempted to breathe spontaneously and were kept in a 28 °C incubator overnight. 48 hrs after the ischemic injury, hearts and plasma were harvested. The hearts were assessed for infarct size as described above. Plasma samples were as analysed for EETs and DHETs as described below.

### 2.9 Quantification of epoxyeicosatrienoic acids and dihydroxyeicosatrienoic acids

To extract oxylipins, epoxyeicosatrienoic acids (EETs) and dihydroxyeicosatrienoic acids (DiHETs) from plasma, a sample aliquot of 200 μl was placed in a glass conical tube containing lyophilised antioxidants and deuterated internal standards. Antioxidants triphenylphosphine and butylated hydroxytoluene were added, at a final concentration of 0.2 % v/v. EDTA was also added to a final concentration of 1 % w/v. The internal standards, 14,15-EETd11 and 14,15-diHETd11, were added at final concentrations of 50 ng/ml and 5 ng/ml, respectively, and desiccated prior to the addition of plasma. Each plasma sample was diluted with 300 μl of phosphate buffer pH 7.8. Lipids were prepared using a Folch extraction by adding 2 mL of 2:1 Chloroform:Methanol to the diluted plasma, left at room temperature for 10 min, and vortexed vigorously at regular intervals. The organic phase containing the oxylipins was recovered by phase partition follow centrifugation and 1500 g for 10 min and was dried under a stream of nitrogen. 1 ml of 1 M NaOH was added to the dried lipid extract and incubated at 90 °C for 20 min. The solution was acidified with formic acid, and lipids were recovered with 4 ml of ethyl acetate. The organic phase separated by phase partitioning following centrifugation at 2500*g* for 5 min, dried under a stream of nitrogen, and dissolved in a final volume of 50 μl of methanol.

Separation of EETs and DiHETs was carried out by reverse phase using an Acquity H Class UPLC system (Waters Corporation), configured with a BEH300C18,100 mm × 2.1 mm, 1.7 μm column (Waters Corporation) with a constant flow rate of 0.4 ml/min. Column temperature was set at 40 °C. Mobile phases consisted of deionized water with 0.02 % acetic acid (A) and acetonitrile (B). The initial condition set at 50 % B ramped to 60 % B in 0.1 min, and held for 1 min at 60 % B, followed by a 2 min step gradient to 70 % B, and again 3 min to 80 % B. A negative ion mode was chosen in electrospray ionization mass spectrometry (ESI) for sample analysis. Data were acquired using the High Definition Mass Spectrometer (HDMS) Synapt G2Si qTof, (Waters Corporation), and MassLynx v4.1 acquisition software (Waters Corporation), and analysed using UNIFI 1.8 (Waters Corporation). Quantification of oxylipins was executed by MRM (multireaction monitoring) in negative ion mode using the following precursor ion > fragment ion transitions:

5(6)EET (*m*/*z* 319.5 > 191.2), 5(6)DiHET (*m*/*z* 337.2 > 145.1), 8(9)EET *m*/*z* 319.2 > 155.1), 8(9)DiHET (*m*/*z* 337.2 > 127.1), 11(12)EET (*m*/*z* 319.2 > 167.1), 11(12)DiHET (*m*/*z* 337.2 > 167.1), 14(15)EET (*m*/*z* 319.2 > 219.2), 14(15)DiHET (*m*/*z* 337.2 > 207.1), 14(15)EET-d11 (*m*/*z* 330.5 > 219.2), 14(15)DiHET-d11 (*m*/*z* 348.6 > 207.1).

EETs were quantified by linear regression analysis following the generation of a standard curve containing all EET analytes at concentrations ranging from 0.039 ng/ml to 50 ng/ml, and a fixed amount of EET d11, the internal standard, at a concentration of 50 ng/ml. Similarly, DiHET were quantified using a standard with calibrants in concentrations ranging from 0.039 pg/ml to 5 ng/ml, and the diHET d11 as an internal standard at a fixed concentration of 5 ng/ml.

### 2.10 DTNB assay

A standard Ellman's assay was used to determine total thiols in samples. Briefly, 10 μl of 10 mM cysteine or RLC14 was incubated with 10 μl of 10 mM 5,50 -dithiobis(2-nitrobenzoic acid) (DTNB) in a final volume of 1 ml with 25 mM HEPES buffer pH 8.5. Absorbance of assay mixtures was measured at 412 nm. In some experiments, TNB (2-Nitro-5-sulfanylbenzoic acid) was used.

### 2.11 LC-MS/MS analysis

For determination of potential adduction of RLC14 to free cysteine, mass spectrometry (MS) and tandem mass spectrometry (MS/MS) analyses were performed in a QExactive Orbitrap system (Thermo Fisher Scientific) equipped with an electrospray (ESI) source. The samples were introduced into the mass spectrometer at a 3 μl/min flow rate using an integrated pump and a 1000 μl volume Hamilton syringe. The spray voltage was set at 3500 V, and nitrogen was used as sheath gas and auxiliary gas at a flow rate of 10 and 1 (arbitrary units), respectively. The source temperature was fixed at 275 °C. Mass Resolution was 35000 at m/z 200 (25000 at m/z 400) and data were obtained in negative mode. MS data were acquired in full scan mode for 0.5 min in the *m*/*z* range 50–1000. MS/MS fragments were obtained in SIM mode for the ions of interest, with collision energies (CE) ranging from 15 to 35 V. Thermo Freestyle application (Thermo Fisher Scientific) was used to view and analyse the acquired spectra.

In separate experiments, sEH protein (4 μg) was denatured with 8 M urea and 0.5 % sodium deoxycholate (SDC) which was then diluted to 1.2 M urea and the protein was incubated overnight at 37 °C with trypsin then acidified with trifluoroacetic acid (TFA) to 1 %. The resulting digested peptide solution was loaded onto an activated (400 μl 50 % acetonitrile (ACN)) and equilibrated (400 μl 5 % ACN with 0.5 % TFA) Pierce C18 spin column (Thermo Scientific) and washed (600 μl 5 % ACN with 0.5 % TFA). Bound peptides were then eluted (40 μl 70 % ACN) and subsequently dried in a vacuum concentrator (Eppendorf). Peptides were resuspended in 5 % ACN with 0.5 % TFA (25 μl).

LC-MS/MS analysis was carried out on a Waters Acquity M-Class UHPLC coupled to a SCIEX ZenoTOF 7600 mass spectrometer. Peptides were first trapped on a Luna C18 micro trap column (5 μm, 100 Å, 20 mm × 0.3 mm, Phenomenex) then separated on a Kinetex XB C18 column (2.6 μm, 100 Å, 150 mm × 0.3 mm, Phenomenex) at 30 °C. The LC sample manager was held at 4 °C and 5 μl of peptide was applied to the column. A 45 min gradient was used for the analysis of peptides. The method employed a flow rate of 6 μl/min and the gradients were developed over the column from 97 % mobile phase A (water with 0.1 % formic acid), 3 % mobile phase B (acetonitrile with 0.1 % formic acid) to 20 % mobile phase A, 80 % mobile phase B. Mass spectrometry data were acquired in positive, data dependent acquisition (DDA) mode. For detailed acquisition parameters, see supplementary information.

LC-MS/MS Data Analysis: DDA data were acquired using SCIEX OS (version 3.4.5.828) with analysis of the raw data performed using MaxQuant (version 2.6.7.0) [37] supplied with UniProt FASTA database (Human UP000005640_9606.fasta) with RLC14 treated as a variable modification. This data analysis was then manually verified using Explorer and BioToolKit within SCIEX OS (version 3.4.5.828). Mass spectrometry figures were produced using GraphPad Prism (version 10.6.1) and Inkscape (version 1.4.2).

## 3 Statistics

Differences between developed pressures were monitored following ischemia and significance was determined using area under the curve. Differences between groups were assessed using ANOVA when ≥3 groups were compared, followed by Tukey post-hoc test or a Student *t*-test when only 2 groups were tested. Differences were considered significant at the 95 % confidence level (P < 0.05).

## 4 Results

### 4.1 NO_2_-OA offers cardioprotection by via C521S-dependent inhibition of sEH

**Fig. 1:**
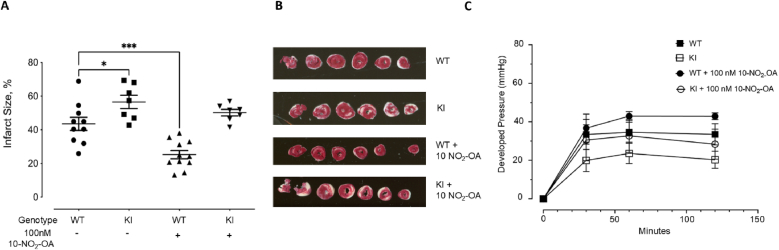
Differential responses of perfused hearts from WT and Cys521Ser KI mice following IR protocol. Mouse hearts were subjected to 30 min global ischemia and 120 min of reperfusion. Following IR protocol, hearts were stained with 2,3,5-triphenyltetrazolium chloride, and infarct size was measured as the % of the area. (**A**) Basally, the KI hearts had a significantly bigger infarct following IR protocol as compared to WT hearts. Perfused hearts from WT and KI mice were subjected to IR injury with or without 100 nM NO_2_-OA treatment for 20 min prior to ischemia. NO_2_-OA significantly decreased the infarct size in WT hearts following the IR protocol, this effect was absent in the KI hearts. In the graph, infarct is quantified, with individual data points for each condition shown on the *left* and the means ± S.E. on the *right* (*n* ≥ 6). (**B**) Representative images of TTC stained WT and C521S KI hearts after IR protocol. TTC stains viable heart muscle deep red while areas of infarction are pale. (**C**) There was an increase in cardiac contractile function during reperfusion, as measured by rate pressure product, following 100 nM NO_2_-OA treatment in WT hearts but in KI hearts.

The C521S KI hearts had a significantly bigger infarct as compared to WT hearts (Fig. 1A and B). There were no significant differences in cardiac developed pressure between WT and KI at reperfusion (Fig. 1C). NO_2_-OA caused a significant decrease in infarct size in WT mice following 20 min perfusion prior to global ischemia (Fig. 1A and B). Although not statistically significant, there was a trend for NO_2_-OA perfusion to increase developed pressure following IR in WT mice. This cardioprotection, in terms of decreased infarct size as well as improvements in contractile function, was not observed in the KI mice.

### 4.2 Inhibitor screen

**Fig. 2:**
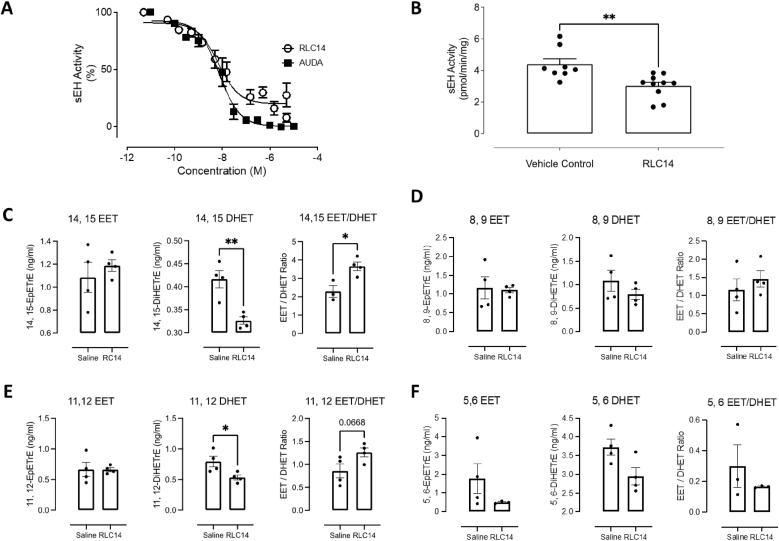
(**A**) IC_50_ for RLC14-dependent inhibition of hydrolase activity. Data was normalised to percentage of control. Also shown is IC_50_ for AUDA. (**B**) sEH activity was assessed in hearts from C57/BL6 mice that had been implanted with an osmotic pump containing RLC14 or saline. RLC14 significantly reduced sEH activity as compared to saline treated mice. (**C–F**) There was a significant decrease in the EET/DHET ratio for 14,15-EET/DHET and non-significant decrease 11,12-EET/DHET as compared to saline treatment. No significant differences were observed for the 8,9- or 5,6-EET/DHET ratios.

We examined 524 potentially electrophilic compounds for their ability to inhibit sEH. The compound library was tested using a sEH inhibitor screening assay in a 96-well plate format to allow the efficient and rapid screening of the compounds. All compounds were first tested at a concentration of 10 μM initially to rapidly rule out compounds with no inhibitory activity. Twenty compounds (Table S1) were analysed further to determine their IC_50_ and we found that STOCK1N-36489 had the lowest IC_50_ value of 6.8x10^−9^ M (Fig. 2A). STOCK1N-36489, which we subsequently refer to as RLC14, was then tested for its ability to protect hearts from injury during IR.

RLC14 or vehicle control was delivered systemically using osmotic mini pumps to WT mice at 0.5 mg/kg for 7 days and the impact on cardiac sEH activity was determined. RLC14 administration significantly reduced sEH activity by 25 % compared to vehicle control (Fig. 2B). Consistent with inhibition of cardiac sEH in mice exposed to RLC14, there was a significant increase in the 14,15-EET/DHET ratio and an increase in the 11,12-EET/DHET ratio in their plasma compared to those that received vehicle alone (Fig. 2C–F). These results are consistent with RLC14 working effectively as an sEH inhibitor *in vivo*.

### 4.3 Assessing cardioprotection against ischemia and reperfusion injury by RLC14

**Fig. 3:**
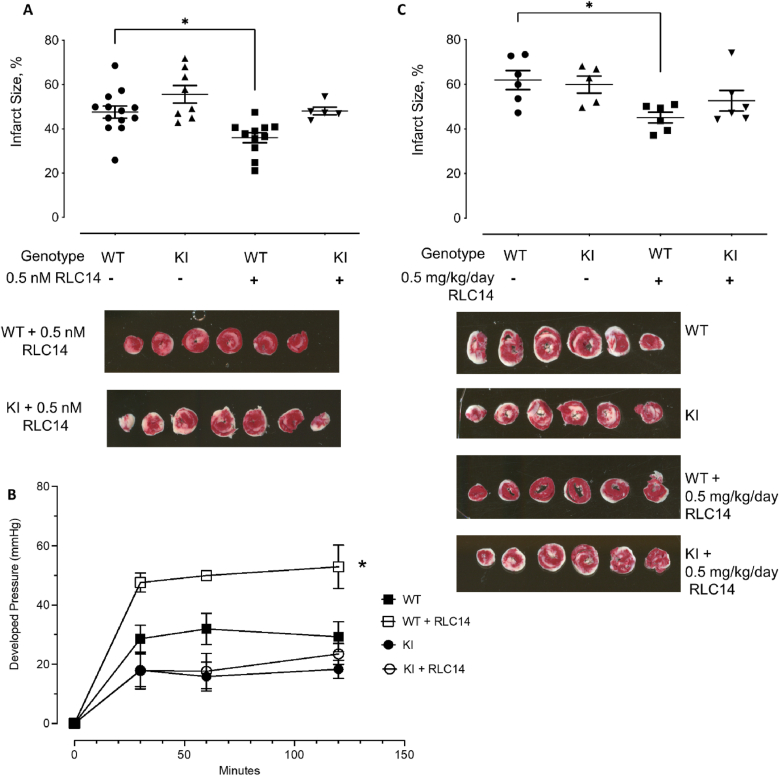
(**A**) Perfused hearts from WT and C521S sEH KI mice were subjected to IR injury with or without 0.5 nM RLC14 treatment for 20 min prior to ischemia. RLC14 treatment significantly decreased the infarct size in WT hearts following the IR protocol, this effect was absent in the KI hearts. In the graph, infarct is quantified, with individual data points for each condition shown on the *left* and the means ± S.E. on the *right* (*n* ≥ 5). Representative images of TTC stained WT and C521S KI 0.5 nM RLC14 treated hearts after IR protocol are shown below. (**B**) There was an increase in cardiac contractile function during reperfusion, as measured by rate pressure product, following 0.5 nM RLC14 treatment in WT hearts but in KI hearts. (**C**) C521S sEH WT and KI mice were subjected to IR injury with or without 0.5 mg/kg/day RLC14 treatment for 7 days prior to ischemia. In the graph, infarct is quantified, with individual data points for each condition shown on the *left* and the means ± S.E. on the *right* (*n* ≥ 6). Representative images of TTC stained WT and C521S KI ± 0.5 mg/kg/day RLC14 treated hearts after IR protocol are shown below.

Isolated perfused hearts administered with 0.5 nM RLC14 for 20 min prior to IR showed a significant decrease in infarct size in WT mice, which was not observed in the C521S sEH KI mice (Fig. 3A). This treatment with 0.5 nM RLC14 also significantly improved cardiac contractile function after IR in WT mice, but this protection was not afforded to the KI mice (Fig. 3B).

To determine if RLC14 treatment was similarly cardioprotective *in vivo*, the drug was delivered systemically to mice for 1 week prior to an IR protocol. Administering RLC14 reduced infarct size caused by IR from 61.9 % to 45 % in WT mice, but this was not observed in KI mice (Fig. 3C), indicating that RLC14 is cardioprotective and does so by adducting to Cys521 of sEH.

### 4.4 Mechanism of adduction to thiols by RLC14

**Fig. 4:**
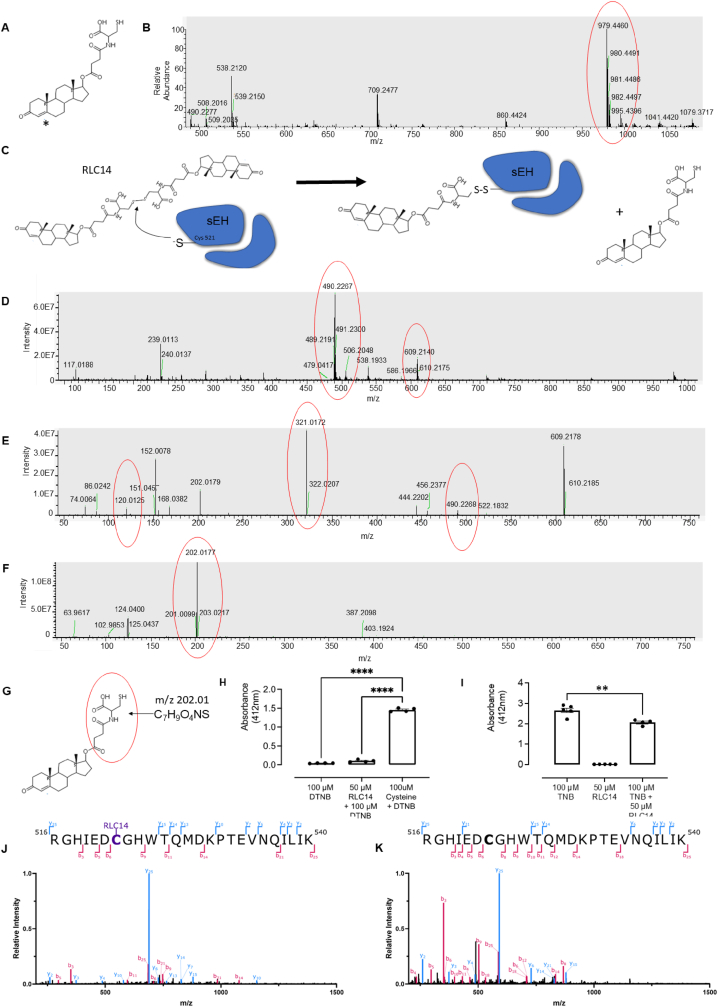
(**A**) Structure of RLC14 as initially proposed. The α,β-unsaturated carbonyl group is designated with an ∗, this group is electrophilic, however due to the ring at the carbon 4 position, the reaction may be hindered. (**B**) The spectral scan of RLC14 shows the presence of an ion at *m*/*z* 979.44 (negative mode), this is consistent with RLC14 existing as a disulfide dimer. (**C**) Outline of how RLC14 adducts to sEH at Cys 521 via disulfide exchange mechanism. (**D**) Full spectral scan of sample containing RLC14 mixed with cysteine, the large, circled area shows the molecular ion of the drug in negative ionization (*m*/*z* 490.2266). A peak consistent with the formation of a disulfide bond between RLC14 and cysteine was evident at *m*/*z* 609.21. (**E**) The ion at *m*/*z* 609.21 was isolated and subsequently fragmented. The resulting ions include ions that correspond to cysteine (*m*/*z* 120.0125) and to RLC14 (*m*/*z* 490.2266). The most abundant ion is *m*/*z* 321.01, which is not present in the earlier spectras, suggesting that it was produced by the fragmentation of the ion *m*/*z* 609.21. (**F**) In order to assess this ion at *m*/*z* 321.01, the molecular ion of the drug (*m*/*z* 490.2266) was also isolated and fragmented with a similar collision energy. After fragmentation, the most abundant ion obtained was *m*/*z* 202.01, which was also present in the fragmentation of RLC14 plus cysteine. The ion is consistent with the chemical formula C_7_H_9_O_4_NS, which was obtained when fragmenting RLC14 as shown in (**G**). The sum of this ion (*m*/*z* 202.01) plus a residue of cysteine bonded by a disulfide bond is *m*/*z* 321.01 which confirming that RLC14 was able to adduct to cysteine residues via the free SH group. (**H–I**) To assess thiol reactivity of RLC14 it was incubated with DTNB, RLC14 showed no thiol reactivity with DTNB, in direct contrast to cysteine. However, when RLC14 was incubated with TNB, there was a significant decrease in absorbance at 412 nm. (**J**) A LC-MS/MS spectra identifying a precursor in the 5+ charge state (688.3421 *m*/*z*) corresponding to a mass of 3436.6741 Da was assigned to the peptide ^516^RGHIEDC[+RLC14]GHWTQMDKPTEVNQILIK^540^ in a RLC14 drug treated protein sample. RLC14 was adducted to cysteine 522 as shown in purple. The peptide was confidently assigned through the identification of its b and y fragment ions as shown. (**K**) A precursor in the 5+ charge state (590.4989 *m*/*z*) corresponding to a mass of 2947.4581 Da was assigned to peptide ^516^RGHIEDCGHWTQMDKPTEVNQILIK^540^, indicating the absence of RLC14 adducted to C522 in the untreated sample.

Initially we thought that RLC14 contained a free thiol moiety, but as explained below, further analysis showed it was a disulfide, but also contained an α,β-unsaturated carbonyl group, which is potentially electrophilic as shown in Fig. 4A. However, because of the ring at the carbon 4 position, the reaction with thiols at this site may be hindered. Hence, how this compound adducts to sEH C521 to elicit cardioprotection was unclear. To investigate the mechanism of adduction, we used tandem mass spectrometry (MS/MS). We analysed RLC14 alone and observed an ion at *m*/*z* 979.44 in negative mode, which is twice the anticipated molecular weight and consistent with RLC14 existing as a disulfide dimer (Fig. 4B). It is therefore likely that RLC14 adducts and inhibits sEH via a C521 thiol-disulfide exchange mechanism (Fig. 4C). Consequently, we looked for evidence of RLC14 adducting to cysteine via disulfide bond formation. When RLC14 was incubated with cysteine we observed molecular ions corresponding to RLC14 (*m*/*z* 490.23) and RLC14-cysteine (*m*/*z* 609.21) (Fig. 4D). To determine if the ion at *m*/*z* 609.21 was indeed a disulfide between cysteine and the drug, it was isolated and fragmented in MS/MS (Fig. 4E). The resulting spectral scan revealed molecular ions that corresponded to cysteine (*m*/*z* 120.01) and to RLC14 (*m*/*z* 490.22). Interestingly, the most abundant ion was observed at *m*/*z* 321.01, which was not present in any of the earlier spectra and from fragmentation of the ion *m*/*z* 609.21. We speculated that the ion at *m*/*z* 321.01 results from fragmentation and is part of RLC14 plus a cysteine residue. To investigate this, the molecular ion of the drug (*m*/*z* 490.2266) was also isolated and fragmented with a comparable collision energy used in the adduct analyses. After fragmentation of the drug, the most abundant ion obtained was *m*/*z* 202.01 (Fig. 4F), this is consistent with the chemical formula C_7_H_9_O_4_NS, which is equivalent to part of RLC14 (Fig. 4G). The sum of this ion (*m*/*z* 202.01) plus a residue of cysteine bonded by a disulfide bond is *m*/*z* 321.01, corroborating our earlier findings in Fig. 4E. Overall these results substantiate that RLC14 adducts to cysteine residues via disulfide bond formation. When RLC14 was mixed with DTNB it showed no thiol reactivity, in direct contrast to cysteine which significantly increased the absorbance at 412 nm (Fig. 4H). In addition, when RLC14 was incubated with TNB thiolate, which is reduced DTNB and responsible for the absorbance at 412 nm, we observed a significant decrease in absorbance at this wavelength (Fig. 4I).

To investigate whether RLC14 forms an adduct with C522 (C521 in mice) in human sEH, full-length recombinant sEH was incubated with or without RLC14. Each sample was then denatured and subsequently digested with trypsin and analysed by LC-MS/MS. In the RLC14-treated sample, peptides containing C522 modified by RLC14 were detected (Fig. 4J). These modifications were confirmed by the expected mass-to-charge (*m/z*) values and by the identification of the corresponding b and y fragment ions. No such adducts were observed in the untreated sEH, demonstrating that the modification occurs specifically when RLC14 reacts with C522 (Fig. 4K). Additionally, RLC14 was found to form adducts at other cysteine residues within the hydrolase domain of sEH, including C232, C423, and C477, notably, these adducts, like that at C521, were similarly reversed by TCEP treatment (Data not shown).

These results together indicate RLC14 is a disulfide dimer that is capable of undergoing disulfide exchange with cysteine thiols, including C521 in sEH, causing inhibition of hydrolase activity.

## 5 Discussion

Lipid electrophiles, which can react with select protein thiols, covalently adduct to cysteine 521 of sEH to inhibit its hydrolase activity [33,34]. This was considered important as pharmacological sEH inhibitors offer broad cardiovascular protection and are being developed by pharmaceutical companies. Thus, lipid electrophiles may serve as endogenous inhibitors of sEH, potentially protecting the cardiovascular system. To investigate this, a transgenic mouse in which the C521 in sEH was systemically replaced by a serine was generated, making this mutant resistant to inhibition by endogenously derived or exogenously applied lipid electrophiles. We found these transgenics were deficient in their BP-lowering response to nitro-oleic acid, an electrophile that is formed endogenously when oleic acid is orally consumed together with nitrite [34]. This provided robust proof that lipid electrophiles do indeed adduct and inhibit sEH, resulting in accumulation of the EET substrates, which are known to couple to BP-lowering. However, as classical sEH inhibitors, such as AUDA, are known to be cardioprotective we questioned whether this electrophilic adduction mechanism of inhibition could also be exploited to elicit cardioprotection.

Basally, in the absence of an intervention, the C521S sEH KI mice are hypertensive as compared to WT littermate controls [34]. Similarly, in this study we observed that infarct size in isolated, buffer-perfused hearts, were larger in the KI mouse and were associated with poorer contractile recovery than WT after being subjected to IR. This protection from IR injury in WT mice is consistent with sEH being expressed in cardiac myocytes and its pharmacological inhibition therein limiting injury [[25], [26], [27], [28], [29], [30]]. Treatment with NO_2_-OA prior to ischemia significantly reduced infarction in WT mice but failed to do so in the KI, indicating that NO_2_-OA provides protection by inhibiting sEH. This observation is consistent with the findings of Nadtochiy et al., who also found that NO_2_-OA was cardioprotective following IR injury [38].

Despite the wealth of evidence showing the therapeutic actions of sEH inhibitors, including for the cardiovascular system, none have succeeded commercially. As such, we sought to identify other inhibitors that target sEH by the same mechanism as endogenous inhibitors such as 10NO_2_-OA. As outlined above, we screened a library of natural electrophilic compounds, with anticipated thiol-oxidation properties. It is important to note, that in a previous study [35], electrophilic lipids were also shown to inhibit sEH by adduction at C423. As, this cysteine is only conserved in primates we are unable to test this in our current mouse models, however, it is conceivable that in humans these novel electrophilic inhibitors may also adduct to C423 and further potentiate sEH inhibition.

Consistent with our objective, a number of electrophilic compounds that inhibited sEH were identified by the hydrolase activity screen. As only 20 compounds inhibited sEH competently, this indicates there is selectivity in the compounds that inhibit sEH despite the library being one of thiol-reactive electrophiles. One compound, RLC14, which had the lowest IC_50_ value of 6.8x10^−9^ M, not only inhibited hydrolase activity in heart tissue but also increased 14,15-EET/DHET and 11,12-EET/DHET ratios in the plasma of mice it was administered to. These data are consistent with RLC14 adducting and inhibiting sEH at C521. RLC14 pre-treatment prior to ischemia not only significantly reduced infarction, but also improved cardiac contractile function during post-ischemic reperfusion. This cardioprotection was completely absent in the KI. Similarly, in mice subjected to myocardial IR *in vivo*, RLC14 treatment significantly reduced infarct size in WTs but failed to do so in KIs that lack the cysteine to which the electrophile adducts to inhibit the hydrolase. In the *ex vivo* model of IR, hearts from KI mice had a significantly larger infarct following IR than those from WT. In contrast, however, when IR was performed *in vivo* the KI mice had a similar infarct size to that of the WT mice. This disparity likely reflects differences between the two models; the *ex vivo* model involves global ischemia while the *in vivo* model is considered less severe with only the LAD being occluded. Isolated *ex vivo* hearts also lacks sympathetic and parasympathetic innervation present in the *in vivo* scenario. Furthermore, the isolated heart is also perfused with Kreb's buffer, rather than blood, which lacks a number of physiological stimuli, such as insulin, which may affect the efficiency of the heart. The *in vivo* heart will also mount an immune response post infarct, which is largely absent in the *ex vivo* model. Despite these differences, both models indicate that the cardioprotection afforded is via RLC14 against IR injury is mediated by adduction to C521 within the hydrolase domain of sEH to cause its inhibition.

When trying to understand the mechanism of electrophilic inhibition, complexity was identified. Indeed, analyses with MS/MS together with DTNB and TNB showed that the drug was in fact a disulfide dimer and did not contain a reduced thiol, as shown in Fig. 4A. Indicating that RLC14 can undergo disulfide exchange reaction with other thiol groups (Fig. 4C). Regardless, further proteomic MS analyses demonstrated that the drug does adduct to C522 (C521 in mouse sequence), confirming our earlier mouse studies. Importantly, because RLC14 adducts via a disulfide bond, it is likely that this may be reversed *in vivo* under reducing conditions such as those maintained by glutathione and thioredoxin systems.

In conclusion, a number of electrophilic molecules that potently inhibit sEH by adducting to C521 have been identified. One of these, which we named RLC14, inhibits sEH *in vivo* in WT mice and this protects them from injury arising from myocardial infarction resulting from ischemia. The inability of RLC14 to reduce infarct in C521S KI mice is consistent with this compound inhibiting hydrolase activity by covalently adducting with sEH C521. This study illustrates how a new understanding of the redox regulation of a protein, in this case oxidative inhibition of sEH by endogenous electrophiles [34,39], can be leveraged for therapeutic drug development. Indeed, we previously showed that cellular oxidants induce an activating disulfide in protein kinase G Iα and that this lowers blood pressure [40,41], a mechanism that was harnessed in the development of a the electrophilic drug G1 that represents a novel class of anti-hypertensives [42]. An increasing number of proteins are known to be thiol redox regulated, and it is possible that they may also be utilised therapeutically by electrophilic drug development. Traditionally, electrophiles have been avoided in development of pharmacotherapies as they were thought to non-selectively conjugate to numerous proteins. However, more recently, this view has been challenged and we now understand that covalent compounds may offer selectivity and potency benefits over traditional drugs [[43], [44], [45], [46]]. Notably, there are a number of widely used drugs, including penicillin and aspirin [44], which mediate their therapeutic actions via covalent adduction to target proteins, highlighting the renewed potential of covalent inhibitors as powerful and selective therapeutic agents.

## CRediT authorship contribution statement

**Rebecca L. Charles:** Conceptualization, Data curation, Formal analysis, Funding acquisition, Investigation, Methodology, Project administration, Resources, Supervision, Writing – original draft, Writing – review & editing. **Mariana Fernandez-Caggiano:** Conceptualization, Data curation, Formal analysis, Investigation, Writing – review & editing. **Olena Rudyk:** Investigation, Writing – review & editing. **Izaak Tyson-Hirst:** Data curation, Formal analysis, Investigation, Methodology, Writing – review & editing. **Mazdak Ehteramyan:** Investigation, Writing – review & editing. **Christopher H. Switzer:** Conceptualization, Writing – review & editing. **Roberto Buccafusca:** Formal analysis, Investigation, Methodology, Writing – review & editing. **Vinothini Rajeeve:** Formal analysis, Investigation, Methodology, Writing – review & editing. **Katiuscia Bianchi:** Methodology, Writing – review & editing. **Valle Morales:** Formal analysis, Investigation, Methodology, Writing – review & editing. **Andrew J. Finch:** Formal analysis, Investigation, Methodology, Writing – review & editing. **Philip Eaton:** Conceptualization, Formal analysis, Funding acquisition, Investigation, Methodology, Resources, Supervision, Writing – original draft, Writing – review & editing.

## Declaration of competing interest

The authors declare that they have no known competing financial interests or personal relationships that could have appeared to influence the work reported in this paper.
