# Supplementary material for: A novel inhibitor of soluble epoxide hydrolase that adducts C521 is cardioprotective
Source: Redox Biol. 2025 Dec 11;89:103974. doi: 10.1016/j.redox.2025.103974 (PMC12768880; doi:10.1016/j.redox.2025.103974)
Supplement: Multimedia component 1 [file mmc1.docx]

**Supplementary information for**

**A novel inhibitor of soluble Epoxide Hydrolase that adducts C521 is cardioprotective**

**Rebecca L Charles^1*^, Mariana Fernandez-Caggiano^1^, Olena Rudyk^2^, Izaak Tyson-Hirst^3^, Mazdak Ehteramyan^1^, Christopher H Switzer^4^, Roberto Buccafusca^5^,** **Vinothini Rajeeve^3^,** **Katiuscia Bianchi^3^**, **Valle Morales^3^**, **Andrew J Finch^3^, Philip Eaton^1^*.**

^1^ William Harvey Research Institute, Faculty of Medicine and Dentistry, Queen Mary University of London, London, UK.

^2^ King's College London British Heart Foundation Centre, School of Cardiovascular and Metabolic Medicine & Sciences, London, UK.

^3^ Barts Cancer Institute, John Vane Science Centre, Queen Mary University of London, London, UK.

^4^ Department of Molecular and Cell Biology, University of Leicester, Leicester, UK.

^5^ School of Biological and Chemical Sciences, Queen Mary University of London, London, UK.

* Correspondence to [r.charles@qmul.ac.uk](mailto:r.charles@qmul.ac.uk) or [p.eaton@qmul.ac.uk](mailto:p.eaton@qmul.ac.uk)

**Author Contributions:** R.L.C., C.H.S., and P.E. designed research; R.L.C., M.F-C., O.R., M.E., V.M., K.B., I.T-H., and R.B. performed research; R.L.C., M.E., R.B., V.M., K.B., I.T-H., A.J.F and P.E. analyzed data; R.L.C. and P.E. wrote the paper.

**This PDF file includes:**

Supplementary Tables 1, 2 & 3

Supplementary proteomic methods.

| Compound | Number | IC_50_ (M) | Compound | Number | IC_50_ (M) |
| --- | --- | --- | --- | --- | --- |
| \|  \| \| --- \| | NAT14-316950 | 8.9x10^-7^ | O  C  H  3  O  O  O  C  H  3  O  C  H  3  C  H  3  H  H  C  H  3 | NAT14-316956 | 3.3x10^-7^ |
| O  C  H  3  N  N  Cl  N  O  N | NAT14-316951 | 1.9x10^-6^ | \|  \| \| --- \| | NAT14-316969 | 3x10^-7^ |
| 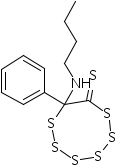 | STOCK1N-14204 | 1.9x10^-6^ | 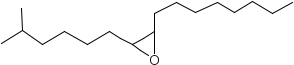 | STOCK1N-00374 | 7.8X10^-7^ |
| 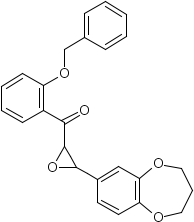 | STOCK1N-01670 | 1.4x10^-6^ | 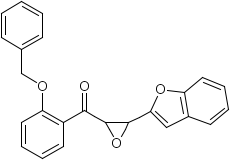 | STOCK1N-06597 | 2.92x10^-6^ |
| 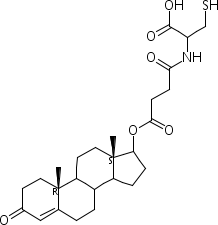 | STOCK1N-36489 (Drug RLC14) ** | 3.5X10^-8^ | 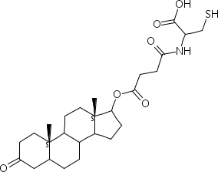 | STOCK1N-43689 | 4.5x10^-7^ |
| 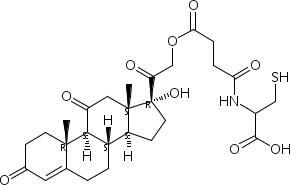 | STOCK1N-55751 | 6.15x10^-7^ | 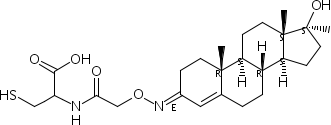 | STOCK1N-56772 | 9.3x10^-7^ |
| 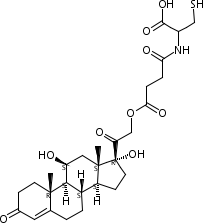 | STOCK1N-56364 | 5.32x10^-7^ | 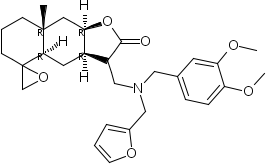 | STOCK1N-47885 | 1.4x10^-7^ |
| 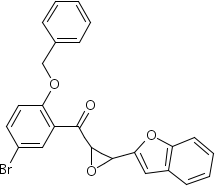 | STOCK1N-03201 | 5.3x10^-6^ | 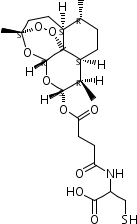 | STOCK1N-57765 | 6.2x10^-7^ |
| 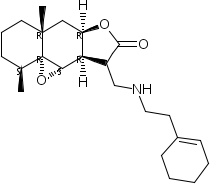 | STOCK1N-33220 | 7.2x10^-7^ | 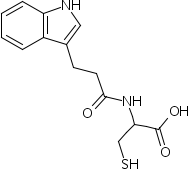 | STOCK1N-62919 | 5.8x10^-8^ |
| 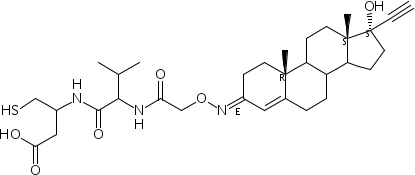 | STOCK1N-36236 | 7.3x10^-7^ | 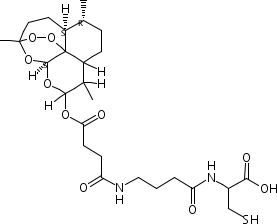 | STOCK1N-64263 | 3.9x10^-7^ |

**Table S1**: IC_50_ value of 20 compounds identified from our compound library as potential sEH inhibitors. ** RLC14 was found to be a disulfide as depicted in Figure 4C in the main manucript.

| **Table S2** | |  |  |  |  |  |
| --- | --- | --- | --- | --- | --- | --- |
| Molecules used in this study | |  |  |  |  |  |
|  |  |  |  |  |  |  |
| 1 | STOCK1N-03030 |  |  |  |  |  |
| 2 | STOCK1N-19321 |  |  |  |  |  |
| 3 | STOCK1N-51902 |  |  |  |  |  |
| 4 | STOCK1N-06800 |  |  |  |  |  |
| 5 | STOCK1N-31035 |  |  |  |  |  |
| 6 | STOCK1N-08486 |  |  |  |  |  |
| 7 | STOCK1N-05758 |  |  | | |  |
| 8 | STOCK1N-07458 |  |  |  |  |  |
| 9 | STOCK1N-03196 |  |  |  |  |  |
| 10 | STOCK1N-57611 |  |  |  |  |  |
| 11 | STOCK1N-31011 |  |  |  |  |  |
| 12 | STOCK1N-73661 |  |  |  |  |  |
| 13 | STOCK1N-08621 |  |  |  |  |  |
| 14 | STOCK1N-06310 |  |  |  |  |  |
| 15 | STOCK1N-06691 |  |  |  |  |  |
| 16 | STOCK1N-04694 |  |  |  |  |  |
| 17 | STOCK1N-11714 |  |  |  |  |  |
| 18 | STOCK1N-03735 |  |  |  |  |  |
| 19 | STOCK1N-55511 |  |  |  |  |  |
| 20 | STOCK1N-16313 |  |  |  |  |  |
| 21 | STOCK1N-06029 |  |  |  |  |  |
| 22 | STOCK1N-54912 |  |  |  |  |  |
| 23 | STOCK1N-57526 |  |  |  |  |  |
| 24 | STOCK1N-58013 |  |  |  |  |  |
| 25 | STOCK1N-05620 |  |  |  |  |  |
| 26 | STOCK1N-56155 |  |  |  |  |  |
| 27 | STOCK1N-24711 |  |  |  |  |  |
| 28 | STOCK1N-71851 |  |  |  |  |  |
| 29 | STOCK1N-49547 |  |  |  |  |  |
| 30 | STOCK1N-09101 |  |  |  |  |  |
| 31 | STOCK1N-54517 |  |  |  |  |  |
| 32 | STOCK1N-09726 |  |  |  |  |  |
| 33 | STOCK1N-46228 |  |  |  |  |  |
| 34 | STOCK1N-44427 |  |  |  |  |  |
| 35 | STOCK1N-16691 |  |  |  |  |  |
| 36 | STOCK1N-13224 |  |  |  |  |  |
| 37 | STOCK1N-06594 |  |  |  |  |  |
| 38 | STOCK1N-09840 |  |  |  |  |  |
| 39 | STOCK1N-24416 |  |  |  |  |  |
| 40 | STOCK1N-01339 |  |  |  |  |  |
| 41 | STOCK1N-01103 |  |  |  |  |  |
| 42 | STOCK1N-14204 |  |  |  |  |  |
| 43 | STOCK1N-12472 |  |  |  |  |  |
| 44 | STOCK1N-03079 |  |  |  |  |  |
| 45 | STOCK1N-11306 |  |  |  |  |  |
| 46 | STOCK1N-43894 |  |  |  |  |  |
| 47 | STOCK1N-45388 |  |  |  |  |  |
| 48 | STOCK1N-05887 |  |  |  |  |  |
| 49 | STOCK1N-05728 |  |  |  |  |  |
| 50 | STOCK1N-06289 |  |  |  |  |  |
| 51 | STOCK1N-11489 |  |  |  |  |  |
| 52 | STOCK1N-51795 |  |  |  |  |  |
| 53 | STOCK1N-04305 |  |  |  |  |  |
| 54 | STOCK1N-03280 |  |  |  |  |  |
| 55 | STOCK1N-11383 |  |  |  |  |  |
| 56 | STOCK1N-11268 |  |  |  |  |  |
| 57 | STOCK1N-03166 |  |  |  |  |  |
| 58 | STOCK1N-57050 |  |  |  |  |  |
| 59 | STOCK1N-57713 |  |  |  |  |  |
| 60 | STOCK1N-30541 |  |  |  |  |  |
| 61 | STOCK1N-73226 |  |  |  |  |  |
| 62 | STOCK1N-18635 |  |  |  |  |  |
| 63 | STOCK1N-07999 |  |  |  |  |  |
| 64 | STOCK1N-10126 |  |  |  |  |  |
| 65 | STOCK1N-06505 |  |  |  |  |  |
| 66 | STOCK1N-67880 |  |  |  |  |  |
| 67 | STOCK1N-02870 |  |  |  |  |  |
| 68 | STOCK1N-57870 |  |  |  |  |  |
| 69 | STOCK1N-28013 |  |  |  |  |  |
| 70 | STOCK1N-24598 |  |  |  |  |  |
| 71 | STOCK1N-44554 |  |  |  |  |  |
| 72 | STOCK1N-06387 |  |  |  |  |  |
| 73 | STOCK1N-05437 |  |  |  |  |  |
| 74 | STOCK1N-08900 |  |  |  |  |  |
| 75 | STOCK1N-06813 |  |  |  |  |  |
| 76 | STOCK1N-00851 |  |  |  |  |  |
| 77 | STOCK1N-06530 |  |  |  |  |  |
| 78 | STOCK1N-04678 |  |  |  |  |  |
| 79 | STOCK1N-09835 |  |  |  |  |  |
| 80 | STOCK1N-07034 |  |  |  |  |  |
| 81 | STOCK1N-57116 |  |  |  |  |  |
| 82 | STOCK1N-23279 |  |  |  |  |  |
| 83 | STOCK1N-52322 |  |  |  |  |  |
| 84 | STOCK1N-27040 |  |  |  |  |  |
| 85 | STOCK1N-30214 |  |  |  |  |  |
| 86 | STOCK1N-46720 |  |  |  |  |  |
| 87 | STOCK1N-43337 |  |  |  |  |  |
| 88 | STOCK1N-55604 |  |  |  |  |  |
| 89 | STOCK1N-42722 |  |  |  |  |  |
| 90 | STOCK1N-24462 |  |  |  |  |  |
| 91 | STOCK1N-16139 |  |  |  |  |  |
| 92 | STOCK1N-15902 |  |  |  |  |  |
| 93 | STOCK1N-23698 |  |  |  |  |  |
| 94 | STOCK1N-22989 |  |  |  |  |  |
| 95 | STOCK1N-24428 |  |  |  |  |  |
| 96 | STOCK1N-16066 |  |  |  |  |  |
| 97 | STOCK1N-16133 |  |  |  |  |  |
| 98 | STOCK1N-16074 |  |  |  |  |  |
| 99 | STOCK1N-24719 |  |  |  |  |  |
| 100 | STOCK1N-24143 |  |  |  |  |  |
| 101 | STOCK1N-22983 |  |  |  |  |  |
| 102 | STOCK1N-15978 |  |  |  |  |  |
| 103 | STOCK1N-16045 |  |  |  |  |  |
| 104 | STOCK1N-15956 |  |  |  |  |  |
| 105 | STOCK1N-60636 |  |  |  |  |  |
| 106 | STOCK1N-20153 |  |  |  |  |  |
| 107 | STOCK1N-18419 |  |  |  |  |  |
| 108 | STOCK1N-18346 |  |  |  |  |  |
| 109 | STOCK1N-18146 |  |  |  |  |  |
| 110 | STOCK1N-52556 |  |  |  |  |  |
| 111 | STOCK1N-19596 |  |  |  |  |  |
| 112 | STOCK1N-18644 |  |  |  |  |  |
| 113 | STOCK1N-21191 |  |  |  |  |  |
| 114 | STOCK1N-19150 |  |  |  |  |  |
| 115 | STOCK1N-20633 |  |  |  |  |  |
| 116 | STOCK1N-18712 |  |  |  |  |  |
| 117 | STOCK1N-19966 |  |  |  |  |  |
| 118 | STOCK1N-43066 |  |  |  |  |  |
| 119 | STOCK1N-46335 |  |  |  |  |  |
| 120 | STOCK1N-70189 |  |  |  |  |  |
| 121 | STOCK1N-45354 |  |  |  |  |  |
| 122 | STOCK1N-41727 |  |  |  |  |  |
| 123 | STOCK1N-44160 |  |  |  |  |  |
| 124 | STOCK1N-47164 |  |  |  |  |  |
| 125 | STOCK1N-42617 |  |  |  |  |  |
| 126 | STOCK1N-68145 |  |  |  |  |  |
| 127 | STOCK1N-54500 |  |  |  |  |  |
| 128 | STOCK1N-67126 |  |  |  |  |  |
| 129 | STOCK1N-71298 |  |  |  |  |  |
| 130 | STOCK1N-31311 |  |  |  |  |  |
| 131 | STOCK1N-52625 |  |  |  |  |  |
| 132 | STOCK1N-53959 |  |  |  |  |  |
| 133 | STOCK1N-03926 |  |  |  |  |  |
| 134 | STOCK1N-69096 |  |  |  |  |  |
| 135 | STOCK1N-54241 |  |  |  |  |  |
| 136 | STOCK1N-23407 |  |  |  |  |  |
| 137 | STOCK1N-24052 |  |  |  |  |  |
| 138 | STOCK1N-23387 |  |  |  |  |  |
| 139 | STOCK1N-29232 |  |  |  |  |  |
| 140 | STOCK1N-28930 |  |  |  |  |  |
| 141 | STOCK1N-24697 |  |  |  |  |  |
| 142 | STOCK1N-23553 |  |  |  |  |  |
| 143 | STOCK1N-24227 |  |  |  |  |  |
| 144 | STOCK1N-23949 |  |  |  |  |  |
| 145 | STOCK1N-22652 |  |  |  |  |  |
| 146 | STOCK1N-23355 |  |  |  |  |  |
| 147 | STOCK1N-22664 |  |  |  |  |  |
| 148 | STOCK1N-23842 |  |  |  |  |  |
| 149 | STOCK1N-23931 |  |  |  |  |  |
| 150 | STOCK1N-22702 |  |  |  |  |  |
| 151 | STOCK1N-24439 |  |  |  |  |  |
| 152 | STOCK1N-23040 |  |  |  |  |  |
| 153 | STOCK1N-29531 |  |  |  |  |  |
| 154 | STOCK1N-23573 |  |  |  |  |  |
| 155 | STOCK1N-23677 |  |  |  |  |  |
| 156 | STOCK1N-23484 |  |  |  |  |  |
| 157 | STOCK1N-29279 |  |  |  |  |  |
| 158 | STOCK1N-28434 |  |  |  |  |  |
| 159 | STOCK1N-24233 |  |  |  |  |  |
| 160 | STOCK1N-29099 |  |  |  |  |  |
| 161 | STOCK1N-29501 |  |  |  |  |  |
| 162 | STOCK1N-24327 |  |  |  |  |  |
| 163 | STOCK1N-23542 |  |  |  |  |  |
| 164 | STOCK1N-22804 |  |  |  |  |  |
| 165 | STOCK1N-23301 |  |  |  |  |  |
| 166 | STOCK1N-24768 |  |  |  |  |  |
| 167 | STOCK1N-23662 |  |  |  |  |  |
| 168 | STOCK1N-24765 |  |  |  |  |  |
| 169 | STOCK1N-28774 |  |  |  |  |  |
| 170 | STOCK1N-24006 |  |  |  |  |  |
| 171 | STOCK1N-49811 |  |  |  |  |  |
| 172 | STOCK1N-03225 |  |  |  |  |  |
| 173 | STOCK1N-10795 |  |  |  |  |  |
| 174 | STOCK1N-01204 |  |  |  |  |  |
| 175 | STOCK1N-45544 |  |  |  |  |  |
| 176 | STOCK1N-07902 |  |  |  |  |  |
| 177 | STOCK1N-30669 |  |  |  |  |  |
| 178 | STOCK1N-10062 |  |  |  |  |  |
| 179 | STOCK1N-55476 |  |  |  |  |  |
| 180 | STOCK1N-08511 |  |  |  |  |  |
| 181 | STOCK1N-11569 |  |  |  |  |  |
| 182 | STOCK1N-25913 |  |  |  |  |  |
| 183 | STOCK1N-02920 |  |  |  |  |  |
| 184 | STOCK1N-00374 |  |  |  |  |  |
| 185 | STOCK1N-34963 |  |  |  |  |  |
| 186 | STOCK1N-16515 |  |  |  |  |  |
| 187 | STOCK1N-58824 |  |  |  |  |  |
| 188 | STOCK1N-69156 |  |  |  |  |  |
| 189 | STOCK1N-48711 |  |  |  |  |  |
| 190 | STOCK1N-16916 |  |  |  |  |  |
| 191 | STOCK1N-17002 |  |  |  |  |  |
| 192 | STOCK1N-69281 |  |  |  |  |  |
| 193 | STOCK1N-11987 |  |  |  |  |  |
| 194 | STOCK1N-31799 |  |  |  |  |  |
| 195 | STOCK1N-24171 |  |  |  |  |  |
| 196 | STOCK1N-54580 |  |  |  |  |  |
| 197 | STOCK1N-53017 |  |  |  |  |  |
| 198 | STOCK1N-03201 |  |  |  |  |  |
| 199 | STOCK1N-06597 |  |  |  |  |  |
| 200 | STOCK1N-02536 |  |  |  |  |  |
| 201 | STOCK1N-04799 |  |  |  |  |  |
| 202 | STOCK1N-01670 |  |  |  |  |  |
| 203 | STOCK1N-32340 |  |  |  |  |  |
| 204 | STOCK1N-08346 |  |  |  |  |  |
| 205 | STOCK1N-24639 |  |  |  |  |  |
| 206 | STOCK1N-22781 |  |  |  |  |  |
| 207 | STOCK1N-32824 |  |  |  |  |  |
| 208 | STOCK1N-23868 |  |  |  |  |  |
| 209 | STOCK1N-22736 |  |  |  |  |  |
| 210 | STOCK1N-23960 |  |  |  |  |  |
| 211 | STOCK1N-24180 |  |  |  |  |  |
| 212 | STOCK1N-24217 |  |  |  |  |  |
| 213 | STOCK1N-23209 |  |  |  |  |  |
| 214 | STOCK1N-24307 |  |  |  |  |  |
| 215 | STOCK1N-30243 |  |  |  |  |  |
| 216 | STOCK1N-57429 |  |  |  |  |  |
| 217 | STOCK1N-03819 |  |  |  |  |  |
| 218 | STOCK1N-55935 |  |  |  |  |  |
| 219 | STOCK1N-58685 |  |  |  |  |  |
| 220 | STOCK1N-06127 |  |  |  |  |  |
| 221 | STOCK1N-17282 |  |  |  |  |  |
| 222 | STOCK1N-45238 |  |  |  |  |  |
| 223 | STOCK1N-05864 |  |  |  |  |  |
| 224 | STOCK1N-55160 |  |  |  |  |  |
| 225 | STOCK1N-53724 |  |  |  |  |  |
| 226 | STOCK1N-55131 |  |  |  |  |  |
| 227 | STOCK1N-33371 |  |  |  |  |  |
| 228 | STOCK1N-33430 |  |  |  |  |  |
| 229 | STOCK1N-33595 |  |  |  |  |  |
| 230 | STOCK1N-24104 |  |  |  |  |  |
| 231 | STOCK1N-33183 |  |  |  |  |  |
| 232 | STOCK1N-33088 |  |  |  |  |  |
| 233 | STOCK1N-33149 |  |  |  |  |  |
| 234 | STOCK1N-33153 |  |  |  |  |  |
| 235 | STOCK1N-36219 |  |  |  |  |  |
| 236 | STOCK1N-33587 |  |  |  |  |  |
| 237 | STOCK1N-33432 |  |  |  |  |  |
| 238 | STOCK1N-24422 |  |  |  |  |  |
| 239 | STOCK1N-24484 |  |  |  |  |  |
| 240 | STOCK1N-24117 |  |  |  |  |  |
| 241 | STOCK1N-32769 |  |  |  |  |  |
| 242 | STOCK1N-33184 |  |  |  |  |  |
| 243 | STOCK1N-33546 |  |  |  |  |  |
| 244 | STOCK1N-32829 |  |  |  |  |  |
| 245 | STOCK1N-24362 |  |  |  |  |  |
| 246 | STOCK1N-33220 |  |  |  |  |  |
| 247 | STOCK1N-23822 |  |  |  |  |  |
| 248 | STOCK1N-32899 |  |  |  |  |  |
| 249 | STOCK1N-32624 |  |  |  |  |  |
| 250 | STOCK1N-58358 |  |  |  |  |  |
| 251 | STOCK1N-32659 |  |  |  |  |  |
| 252 | STOCK1N-36236 |  |  |  |  |  |
| 253 | STOCK1N-55751 |  |  |  |  |  |
| 254 | STOCK1N-36489 |  |  |  |  |  |
| 255 | STOCK1N-56364 |  |  |  |  |  |
| 256 | STOCK1N-55287 |  |  |  |  |  |
| 257 | STOCK1N-41046 |  |  |  |  |  |
| 258 | STOCK1N-39260 |  |  |  |  |  |
| 259 | STOCK1N-37674 |  |  |  |  |  |
| 260 | STOCK1N-37611 |  |  |  |  |  |
| 261 | STOCK1N-38334 |  |  |  |  |  |
| 262 | STOCK1N-38055 |  |  |  |  |  |
| 263 | STOCK1N-23122 |  |  |  |  |  |
| 264 | STOCK1N-37692 |  |  |  |  |  |
| 265 | STOCK1N-49875 |  |  |  |  |  |
| 266 | STOCK1N-51883 |  |  |  |  |  |
| 267 | STOCK1N-44600 |  |  |  |  |  |
| 268 | STOCK1N-43527 |  |  |  |  |  |
| 269 | STOCK1N-60515 |  |  |  |  |  |
| 270 | STOCK1N-41828 |  |  |  |  |  |
| 271 | STOCK1N-43689 |  |  |  |  |  |
| 272 | STOCK1N-54271 |  |  |  |  |  |
| 273 | STOCK1N-52221 |  |  |  |  |  |
| 274 | STOCK1N-51453 |  |  |  |  |  |
| 275 | STOCK1N-53538 |  |  |  |  |  |
| 276 | STOCK1N-53842 |  |  |  |  |  |
| 277 | STOCK1N-52359 |  |  |  |  |  |
| 278 | STOCK1N-50257 |  |  |  |  |  |
| 279 | STOCK1N-49221 |  |  |  |  |  |
| 280 | STOCK1N-52388 |  |  |  |  |  |
| 281 | STOCK1N-51846 |  |  |  |  |  |
| 282 | STOCK1N-50674 |  |  |  |  |  |
| 283 | STOCK1N-53036 |  |  |  |  |  |
| 284 | STOCK1N-48032 |  |  |  |  |  |
| 285 | STOCK1N-51821 |  |  |  |  |  |
| 286 | STOCK1N-52862 |  |  |  |  |  |
| 287 | STOCK1N-54075 |  |  |  |  |  |
| 288 | STOCK1N-50018 |  |  |  |  |  |
| 289 | STOCK1N-54462 |  |  |  |  |  |
| 290 | STOCK1N-53052 |  |  |  |  |  |
| 291 | STOCK1N-51383 |  |  |  |  |  |
| 292 | STOCK1N-50855 |  |  |  |  |  |
| 293 | STOCK1N-51705 |  |  |  |  |  |
| 294 | STOCK1N-52753 |  |  |  |  |  |
| 295 | STOCK1N-48731 |  |  |  |  |  |
| 296 | STOCK1N-48793 |  |  |  |  |  |
| 297 | STOCK1N-47394 |  |  |  |  |  |
| 298 | STOCK1N-47851 |  |  |  |  |  |
| 299 | STOCK1N-48774 |  |  |  |  |  |
| 300 | STOCK1N-48724 |  |  |  |  |  |
| 301 | STOCK1N-48732 |  |  |  |  |  |
| 302 | STOCK1N-48122 |  |  |  |  |  |
| 303 | STOCK1N-49123 |  |  |  |  |  |
| 304 | STOCK1N-48619 |  |  |  |  |  |
| 305 | STOCK1N-48644 |  |  |  |  |  |
| 306 | STOCK1N-47748 |  |  |  |  |  |
| 307 | STOCK1N-49178 |  |  |  |  |  |
| 308 | STOCK1N-47530 |  |  |  |  |  |
| 309 | STOCK1N-48682 |  |  |  |  |  |
| 310 | STOCK1N-49242 |  |  |  |  |  |
| 311 | STOCK1N-47727 |  |  |  |  |  |
| 312 | STOCK1N-48887 |  |  |  |  |  |
| 313 | STOCK1N-49323 |  |  |  |  |  |
| 314 | STOCK1N-47619 |  |  |  |  |  |
| 315 | STOCK1N-49446 |  |  |  |  |  |
| 316 | STOCK1N-48033 |  |  |  |  |  |
| 317 | STOCK1N-47665 |  |  |  |  |  |
| 318 | STOCK1N-48794 |  |  |  |  |  |
| 319 | STOCK1N-47453 |  |  |  |  |  |
| 320 | STOCK1N-49121 |  |  |  |  |  |
| 321 | STOCK1N-49274 |  |  |  |  |  |
| 322 | STOCK1N-49196 |  |  |  |  |  |
| 323 | STOCK1N-48985 |  |  |  |  |  |
| 324 | STOCK1N-49327 |  |  |  |  |  |
| 325 | STOCK1N-47529 |  |  |  |  |  |
| 326 | STOCK1N-48943 |  |  |  |  |  |
| 327 | STOCK1N-47786 |  |  |  |  |  |
| 328 | STOCK1N-48358 |  |  |  |  |  |
| 329 | STOCK1N-48195 |  |  |  |  |  |
| 330 | STOCK1N-49099 |  |  |  |  |  |
| 331 | STOCK1N-48164 |  |  |  |  |  |
| 332 | STOCK1N-47877 |  |  |  |  |  |
| 333 | STOCK1N-47885 |  |  |  |  |  |
| 334 | STOCK1N-48800 |  |  |  |  |  |
| 335 | STOCK1N-48684 |  |  |  |  |  |
| 336 | STOCK1N-47657 |  |  |  |  |  |
| 337 | STOCK1N-48323 |  |  |  |  |  |
| 338 | STOCK1N-47810 |  |  |  |  |  |
| 339 | STOCK1N-51495 |  |  |  |  |  |
| 340 | STOCK1N-56772 |  |  |  |  |  |
| 341 | STOCK1N-50434 |  |  |  |  |  |
| 342 | STOCK1N-66345 |  |  |  |  |  |
| 343 | STOCK1N-44321 |  |  |  |  |  |
| 344 | STOCK1N-69994 |  |  |  |  |  |
| 345 | STOCK1N-57765 |  |  |  |  |  |
| 346 | STOCK1N-56995 |  |  |  |  |  |
| 347 | STOCK1N-62919 |  |  |  |  |  |
| 348 | STOCK1N-64263 |  |  |  |  |  |
| 349 | STOCK1N-59361 |  |  |  |  |  |
| 350 | STOCK1N-61856 |  |  |  |  |  |
| 351 | STOCK1N-63076 |  |  |  |  |  |
| 352 | STOCK1N-59711 |  |  |  |  |  |
| 353 | STOCK1N-61627 |  |  |  |  |  |
| 354 | STOCK1N-59481 |  |  |  |  |  |
| 355 | STOCK1N-59372 |  |  |  |  |  |
| 356 | STOCK1N-63343 |  |  |  |  |  |
| 357 | STOCK1N-65892 |  |  |  |  |  |
| 358 | STOCK1N-66001 |  |  |  |  |  |
| 359 | STOCK1N-65962 |  |  |  |  |  |
| 360 | STOCK1N-65813 |  |  |  |  |  |
| 361 | STOCK1N-59567 |  |  |  |  |  |
| 362 | STOCK1N-59597 |  |  |  |  |  |
| 363 | STOCK1N-59760 |  |  |  |  |  |
| 364 | STOCK1N-62340 |  |  |  |  |  |
| 365 | STOCK1N-60376 |  |  |  |  |  |
| 366 | STOCK1N-60669 |  |  |  |  |  |
| 367 | STOCK1N-63584 |  |  |  |  |  |
| 368 | STOCK1N-60559 |  |  |  |  |  |
| 369 | STOCK1N-61917 |  |  |  |  |  |
| 370 | STOCK1N-59127 |  |  |  |  |  |
| 371 | STOCK1N-67951 |  |  |  |  |  |
| 372 | STOCK1N-06778 |  |  |  |  |  |
| 373 | STOCK1N-50253 |  |  |  |  |  |
| 374 | STOCK1N-12110 |  |  |  |  |  |
| 375 | STOCK1N-65687 |  |  |  |  |  |
| 376 | STOCK1N-62829 |  |  |  |  |  |
| 377 | STOCK1N-62053 |  |  |  |  |  |
| 378 | STOCK1N-61538 |  |  |  |  |  |
| 379 | STOCK1N-63309 |  |  |  |  |  |
| 380 | STOCK1N-59395 |  |  |  |  |  |
| 381 | STOCK1N-60563 |  |  |  |  |  |
| 382 | STOCK1N-58990 |  |  |  |  |  |
| 383 | STOCK1N-59303 |  |  |  |  |  |
| 384 | STOCK1N-61829 |  |  |  |  |  |
| 385 | STOCK1N-62974 |  |  |  |  |  |
| 386 | STOCK1N-60287 |  |  |  |  |  |
| 387 | STOCK1N-63429 |  |  |  |  |  |
| 388 | STOCK1N-62354 |  |  |  |  |  |
| 389 | STOCK1N-62681 |  |  |  |  |  |
| 390 | STOCK1N-61002 |  |  |  |  |  |
| 391 | STOCK1N-62148 |  |  |  |  |  |
| 392 | STOCK1N-60534 |  |  |  |  |  |
| 393 | STOCK1N-62843 |  |  |  |  |  |
| 394 | STOCK1N-62421 |  |  |  |  |  |
| 395 | STOCK1N-60737 |  |  |  |  |  |
| 396 | STOCK1N-60326 |  |  |  |  |  |
| 397 | STOCK1N-62768 |  |  |  |  |  |
| 398 | STOCK1N-60484 |  |  |  |  |  |
| 399 | STOCK1N-62754 |  |  |  |  |  |
| 400 | STOCK1N-59791 |  |  |  |  |  |
| 401 | STOCK1N-60197 |  |  |  |  |  |
| 402 | STOCK1N-63335 |  |  |  |  |  |
| 403 | STOCK1N-63845 |  |  |  |  |  |
| 404 | STOCK1N-60578 |  |  |  |  |  |
| 405 | STOCK1N-61374 |  |  |  |  |  |
| 406 | STOCK1N-63662 |  |  |  |  |  |
| 407 | STOCK1N-60068 |  |  |  |  |  |
| 408 | STOCK1N-61347 |  |  |  |  |  |
| 409 | STOCK1N-62588 |  |  |  |  |  |
| 410 | STOCK1N-66610 |  |  |  |  |  |
| 411 | STOCK1N-66720 |  |  |  |  |  |
| 412 | STOCK1N-63304 |  |  |  |  |  |
| 413 | STOCK1N-61430 |  |  |  |  |  |
| 414 | STOCK1N-59844 |  |  |  |  |  |
| 415 | STOCK1N-62937 |  |  |  |  |  |
| 416 | STOCK1N-59247 |  |  |  |  |  |
| 417 | STOCK1N-71165 |  |  |  |  |  |
| 418 | NP-009836 |  |  |  |  |  |
| 419 | NP-002901 |  |  |  |  |  |
| 420 | NP-008134 |  |  |  |  |  |
| 421 | NP-001476 |  |  |  |  |  |
| 422 | NP-008410 |  |  |  |  |  |
| 423 | NP-012195 |  |  |  |  |  |
| 424 | NP-012338 |  |  |  |  |  |
| 425 | NP-012854 |  |  |  |  |  |
| 426 | NP-004595 |  |  |  |  |  |
| 427 | NP-003767 |  |  |  |  |  |
| 428 | NP-000960 |  |  |  |  |  |
| 429 | NP-000233 |  |  |  |  |  |
| 430 | NP-003031 |  |  |  |  |  |
| 431 | NP-007847 |  |  |  |  |  |
| 432 | NP-007582 |  |  |  |  |  |
| 433 | NP-002483 |  |  |  |  |  |
| 434 | NP-007749 |  |  |  |  |  |
| 435 | NP-007883 |  |  |  |  |  |
| 436 | NP-004848 |  |  |  |  |  |
| 437 | NP-003649 |  |  |  |  |  |
| 438 | NP-003320 |  |  |  |  |  |
| 439 | NP-008635 |  |  |  |  |  |
| 440 | NP-000831 |  |  |  |  |  |
| 441 | NP-003614 |  |  |  |  |  |
| 442 | NP-009775 |  |  |  |  |  |
| 443 | NP-007072 |  |  |  |  |  |
| 444 | NP-005530 |  |  |  |  |  |
| 445 | NP-012196 |  |  |  |  |  |
| 446 | NP-007851 |  |  |  |  |  |
| 447 | NP-007388 |  |  |  |  |  |
| 448 | NP-008856 |  |  |  |  |  |
| 449 | NP-012386 |  |  |  |  |  |
| 450 | NP-013094 |  |  |  |  |  |
| 451 | NP-006503 |  |  |  |  |  |
| 452 | NP-000129 |  |  |  |  |  |
| 453 | NP-004702 |  |  |  |  |  |
| 454 | NP-000828 |  |  |  |  |  |
| 455 | NP-010981 |  |  |  |  |  |
| 456 | NP-006783 |  |  |  |  |  |
| 457 | NP-002105 |  |  |  |  |  |
| 458 | NP-009825 |  |  |  |  |  |
| 459 | NP-003486 |  |  |  |  |  |
| 460 | NP-004826 |  |  |  |  |  |
| 461 | NP-000484 |  |  |  |  |  |
| 462 | NP-011876 |  |  |  |  |  |
| 463 | NP-003457 |  |  |  |  |  |
| 464 | NP-005534 |  |  |  |  |  |
| 465 | NP-000294 |  |  |  |  |  |
| 466 | NP-013157 |  |  |  |  |  |
| 467 | NP-003824 |  |  |  |  |  |
| 468 | NP-007464 |  |  |  |  |  |
| 469 | NP-009103 |  |  |  |  |  |
| 470 | NP-001328 |  |  |  |  |  |
| 471 | NP-005872 |  |  |  |  |  |
| 472 | NP-014435 |  |  |  |  |  |
| 473 | NP-014343 |  |  |  |  |  |
| 474 | NP-015076 |  |  |  |  |  |
| 475 | NP-002688 |  |  |  |  |  |
| 476 | NP-004323 |  |  |  |  |  |
| 477 | NP-015332 |  |  |  |  |  |
| 478 | NP-015335 |  |  |  |  |  |
| 479 | NP-001229 |  |  |  |  |  |
| 480 | NP-002481 |  |  |  |  |  |
| 481 | NAT14-262548 |  |  |  |  |  |
| 482 | NAT14-262567 |  |  |  |  |  |
| 483 | NAT14-262582 |  |  |  |  |  |
| 484 | NAT14-316946 |  |  |  |  |  |
| 485 | NAT14-316947 |  |  |  |  |  |
| 486 | NAT14-316948 |  |  |  |  |  |
| 487 | NAT14-316949 |  |  |  |  |  |
| 488 | NAT14-316950 |  |  |  |  |  |
| 489 | NAT14-316951 |  |  |  |  |  |
| 490 | NAT14-316956 |  |  |  |  |  |
| 491 | NAT14-316957 |  |  |  |  |  |
| 492 | NAT14-316961 |  |  |  |  |  |
| 493 | NAT14-316962 |  |  |  |  |  |
| 494 | NAT14-316964 |  |  |  |  |  |
| 495 | NAT14-316967 |  |  |  |  |  |
| 496 | NAT14-316968 |  |  |  |  |  |
| 497 | NAT14-316969 |  |  |  |  |  |
| 498 | NAT14-316970 |  |  |  |  |  |
| 499 | NAT14-316971 |  |  |  |  |  |
| 500 | NAT14-316973 |  |  |  |  |  |
| 501 | NAT14-316978 |  |  |  |  |  |
| 502 | NAT14-316981 |  |  |  |  |  |
| 503 | NAT14-322567 |  |  |  |  |  |
| 504 | NAT14-335133 |  |  |  |  |  |
| 505 | NAT14-335413 |  |  |  |  |  |
| 506 | NAT14-335581 |  |  |  |  |  |
| 507 | NAT14-336085 |  |  |  |  |  |
| 508 | NAT14-336141 |  |  |  |  |  |
| 509 | NAT14-336197 |  |  |  |  |  |
| 510 | NAT14-336421 |  |  |  |  |  |
| 511 | NAT14-336589 |  |  |  |  |  |
| 512 | NAT14-336645 |  |  |  |  |  |
| 513 | NAT14-336757 |  |  |  |  |  |
| 514 | NAT14-336869 |  |  |  |  |  |
| 515 | NAT14-346391 |  |  |  |  |  |
| 516 | NAT14-346400 |  |  |  |  |  |
| 517 | NAT14-346401 |  |  |  |  |  |
| 518 | NAT7-254154 |  |  |  |  |  |
| 519 | NAT7-254159 |  |  |  |  |  |
| 520 | NAT7-254160 |  |  |  |  |  |
| 521 | NAT7-254162 |  |  |  |  |  |
| 522 | NAT7-254163 |  |  |  |  |  |
| 523 | NAT7-254167 |  |  |  |  |  |
| 524 | NAT8-264022 |  |  |  |  |  |

**Table S2**: Listed compounds possess potential electrophilic properties and were sourced from AnalytiCon Discovery and InterBioscreen. Highlighted molecules indicate those selected for further detailed analysis.

Supplementary Proteomics LC-MS methods:

| **Time (min)** | **Flow (μl/min)** | **Mobile Phase A (%)** | **Mobile Phase B (%)** |
| --- | --- | --- | --- |
| 0.00 | 6 | 97 | 3 |
| 5.00 | 6 | 97 | 3 |
| 36.00 | 6 | 60 | 40 |
| 37.00 | 6 | 20 | 80 |
| 39.00 | 6 | 20 | 80 |
| 40.00 | 6 | 97 | 3 |
| 45.00 | 6 | 97 | 3 |

**Table S3**: LC–MS gradient profile used for peptide analysis. A 45-minute chromatographic gradient was run at a constant flow rate of 6 μL/min. Mobile phase A consisted of water with 0.1% formic acid, and mobile phase B consisted of acetonitrile with 0.1% formic acid. The gradient was programmed from 97% A / 3% B to 20% A / 80% B over the course of the run, with re-equilibration to initial conditions at the end of the method. The table summarises the time, flow rate, and mobile-phase composition throughout the analytical gradient.

The SCIEX ZenoTOF 7600 was equipped with an OptiFlow Turbo V electrospray ionisation (ESI) source with an OptiFlow 1–50 μL Micro electrode fitted. Data were acquired in positive ion mode with a spray voltage of 5000 V and a source temperature of 300°C. Ion source gas 1 and 2 were set to 20 and 60 psi respectively, with a curtain gas of 35 psi and a CAD gas of 7. For TOF MS scans, a declustering potential (DP) of 80 V and a collision voltage of 10 V were applied, without spread, across a scan range of 400 – 1200 m/z with a 0.1 s accumulation time. For data dependent acquisition (DDA) measurements the maximum number of candidate ions was set to 50 with an intensity threshold of 300 cps. For DDA TOF MSMS scans, Q1 resolution was set to unit, with an accumulation time of 0.01 s, across a scan range of 200 – 1500 m/z. A DP of 80 V was applied, without spread, and a dynamic collision voltage which was dependent on the charge and m/z value of the precursor ions was applied. Zeno pulsing was activated with the threshold set at 100000 cps.
